# Malakoplakia among kidney transplant recipients: case series and literature review

**DOI:** 10.3389/fimmu.2025.1605146

**Published:** 2025-05-23

**Authors:** Ali B. Abbasi, Gerardo Gamino, Alan Zambeli-Ljepović, Adrian M. Whelan, Garrett R. Roll, Peter J. Altshuler

**Affiliations:** ^1^ Department of Surgery, University of California, San Francisco, San Francisco, CA, United States; ^2^ School of Medicine, University of California, San Francisco, San Francisco, CA, United States; ^3^ Department of Medicine, Division of Nephrology, University of California, San Francisco, San Francisco, CA, United States; ^4^ Department of Surgery, Division of Transplantation, University of California, San Francisco, San Francisco, CA, United States

**Keywords:** malakoplakia, kidney transplant, opportunistic infection, immunosuppression, posttransplant management, case report, case series

## Abstract

We report two cases of malakoplakia after kidney transplant, a rare granulomatous condition that occurs primarily in immunocompromised patients and his thought to occur due to incomplete clearance of phagocytized bacterial residue by macrophages. Both patients were at heightened immunological risk due to being highly sensitized or prior episodes of rejection, both experienced *E. Coli* infections in the first 4 months after transplant, and both presented with granulomatous masses that were biopsied and confirmed to be malakoplakia. Both were treated with suppressive antibiotics and required urinary drainage of the transplant kidney, resulting in improvements in the size of the mass on imaging. Given that both patients were at heightened immunological risk due to sensitization or episodes of rejection, we sought to investigate whether these are common risk factors for malakoplakia in the published literature. We summarized 59 published reports of malakoplakia in kidney transplant recipients. We found that malakoplakia cases predominantly occur in the first two years after transplant and that 47% of patients had either prior rejection or a prior transplant. We also found that many case reports of malakoplakia involve *E. Coli* infections and that improvement or resolution of malakoplakia was more common in case reports that did not involve surgical resection of the mass.

## Introduction

Malakoplakia - “soft plaque” in Greek - is a rare condition that involves the development of granulomatous masses or plaques, which can affect any organ system (1). The etiology remains incompletely understood but is hypothesized to be related to incomplete clearance of phagocytized bacterial residue by macrophages, which form enlarged phagolysosomes, known as the characteristic Michaelis-Gutmann bodies (2). Malakoplakia most commonly occurs in patients who are immunocompromised in the setting of organ transplantation, HIV, or malignancy. While malakoplakia can affect any organ system, it is most commonly associated with urinary tract infections (UTIs) due to *Escherichia coli* (*E. coli)*. The rarity of reported cases limits our understanding of specific infectious, immune, or other risk factors (3).

**Figure 1:**
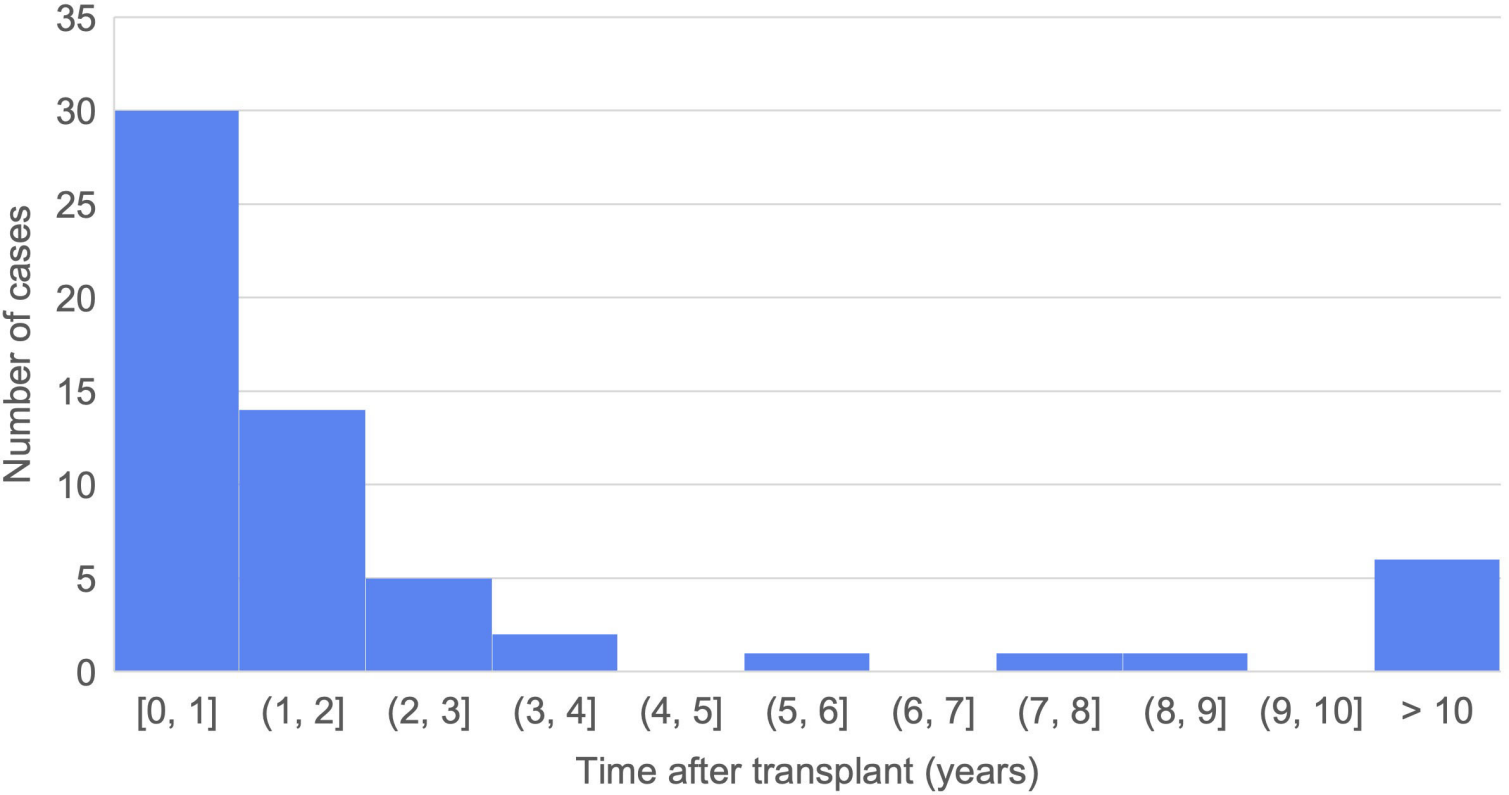
Summary of two cases of malakoplakia.

Here, we describe two cases of malakoplakia after kidney transplantation at our institution, both of which had a need for increased immunosuppression (**Figure 1**). These cases prompted our interest in better characterizing the risk factors for malakoplakia, which remain poorly understood. To this end, we summarized the literature on malakoplakia among kidney transplant recipients by reviewing all published cases in the literature. We characterized potential immunologic and other risk factors for developing the disease and provide clinical correlation to help guide care in patients who develop malakoplakia following kidney transplantation.

## Case presentations

### Case 1

The patient was a 55-year-old woman with end-stage kidney disease (ESKD) due to diabetic nephropathy who underwent a second deceased donor kidney transplant (DDKT) with induction using anti-thymocyte globulin, methylprednisolone, and intravenous immunoglobulin (IVIG) as she was highly sensitized (100% calculated panel reactive antibody, cPRA) with pre-formed donor specific antibodies (DSA). Maintenance immunosuppression was initiated with mycophenolate mofetil, tacrolimus and prednisone. There were no immediate surgical complications, but she was discharged with a foley catheter as her bladder was noted to be very small. Three weeks after transplant, she was readmitted with a catheter- associated. urinary tract infection requiring a 14 day course of IV antibiotics.

Two months post-transplantation she developed a perinephric abscess of the kidney transplant, which was treated with percutaneous drainage. Abscess fluid cultures revealed extended spectrum beta lactamase-producing (ESBL) *E. coli* and *Klebsiella pneumoniae*. She received intravenous ertapenem, but on follow-up imaging two months later was found to have a 5.9x4.9x3.3xcm perinephric, soft tissue mass between the allograft and the iliacus muscle. Biopsy showed Michaelis-Gutmann bodies consistent with malakoplakia. The patient was treated with further drainage, long-term minocycline, and reduction in maintenance immunosuppression.

Two years post-transplantation, she developed an ESBL *E. coli* urinary tract infection and was noted to have hydronephrosis of the kidney transplant. Cystoscopy revealed multifocal tan-white bladder plaques, with biopsy results reconfirming malakoplakia. The hydronephrosis was attributed to mass effect related to the malakoplakia and was managed with percutaneous nephrostomy tube and subsequent conversion to internal stent of the transplant ureter. She was treated with an initial course of IV antibiotics and continued on long-term minocycline.

Three years post-transplantation, the patient remains on minocycline and continues to require a ureteral stent. The mass has decreased in size to 2.6x2.4x1.6cm. Creatinine is stable around 2.0 mg/dL, from a post-transplant nadir around 1.6 mg/dL. The patient reports she has improved quality of life since her kidney transplant and is tolerating antibiotic therapy without issue. She reports no symptoms related to malakoplakia at this time but has needed to make extra trips to the hospital for imaging and management of the ureteric stent.

### Case 2

A 25-year-old man with ESKD of unknown etiology and cPRA 0% underwent DDKT with induction immunosuppression using basiliximab and methylprednisolone. Maintenance immunosuppression was started with mycophenolate mofetil, tacrolimus, and prednisone. There were no immediate surgical complications, but his course was complicated by acute cellular rejection diagnosed four days after transplantation (Banff 1b), which was treated with thymoglobulin and intravenous steroids. Two weeks after transplantation, he was diagnosed with antibody mediated rejection with new DSAs requiring plasmapheresis and IVIG. One month after transplantation, he had an episode of culture-negative epididymoorchitis and two months after transplant he developed hydronephrosis requiring percutaneous nephrostomy (PCN) tube. Three months after transplantation, PCN output became purulent and he was diagnosed with an abdominal wall abscess requiring debridement, with both urine and abscess fluid culture growing *E. coli*. He was discharged on amoxicillin-clavulanic acid with ongoing urinary drainage via PCN.

Four months after transplantation he was found to have a measuring 5.9 x 11.0 x 12.8 cm lobulated soft tissue mass in the right iliac fossa, abutting the transplant ureter and invading the bladder, initially concerning for post-transplant lymphoproliferative disease. Urine cultures grew vancomycin-resistant *Enterococcus*, and a biopsy of the mass was consistent with malakoplakia. Immunosuppression was reduced and he was started on IV ceftriaxone and daptomycin. Six months after transplantation, the mass had reduced in size to 9.3 x 4.4 x 8.5 cm. Nine months after transplantation, he was transitioned to oral cefpodoxime and doxycycline, the PCN was removed, and creatinine was at its post-transplant nadir of 2.5mg/dL. The patient is doing well and reports tolerating IV antibiotics. He is planned to switch to oral therapy after interval imaging.

## Methods: literature review

We performed a review of the literature using the search terms in **Supplementary Table S1** derived from a previous review on this topic (3). We included all case reports of malakoplakia in kidney transplant recipients. From each case, we extracted a standardized dataset, including the involved organ, microorganism, immunologic history, duration of antibiotics, procedures, and clinical outcome. We computed descriptive statistics for these variables.

## Results

**Figure 2:**
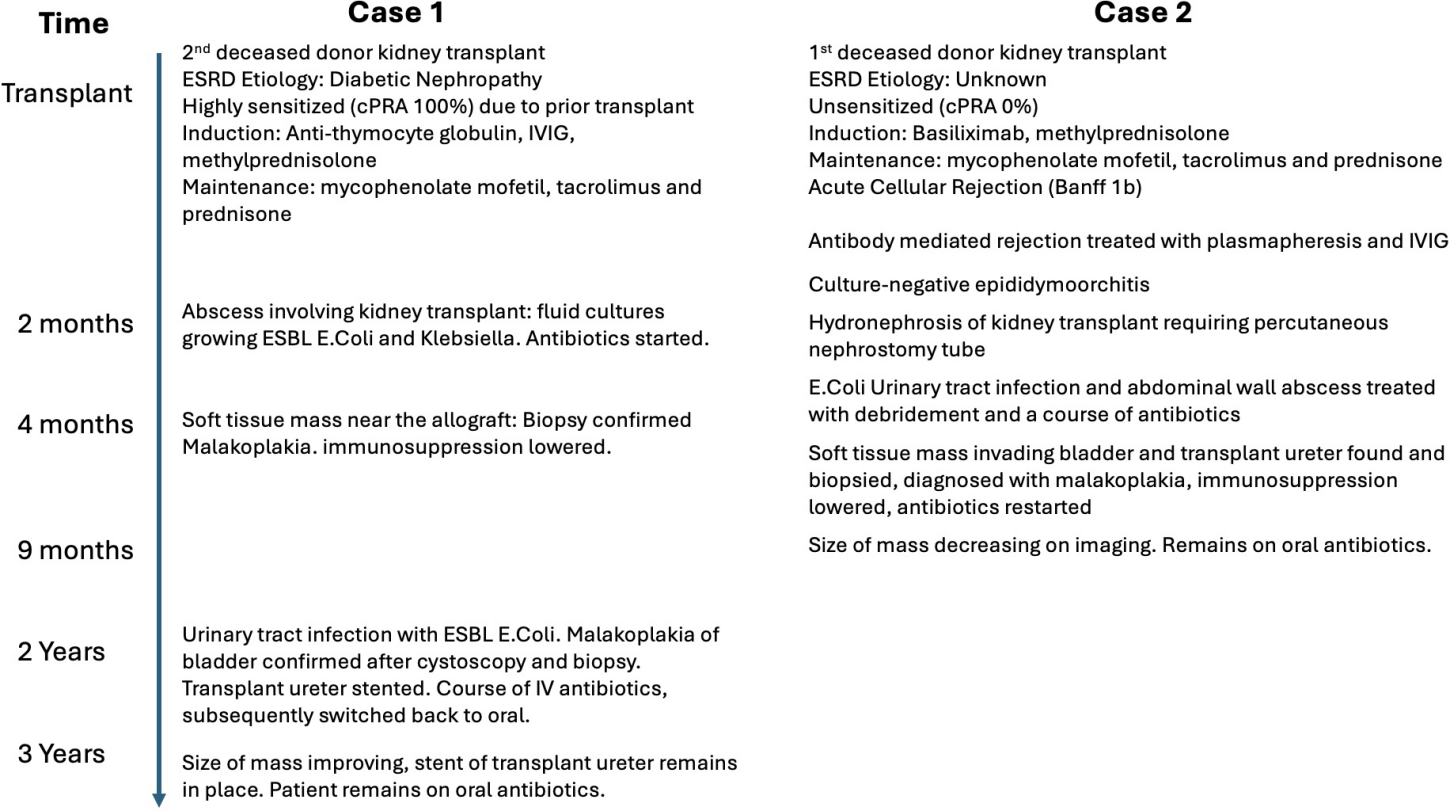
Histogram of time to diagnosis of malakoplakia in years after transplant.

In addition to the two cases we reported above, we identified 57 cases published in the literature (total = 59) (**Table 1**). Malakoplakia was diagnosed within 2 years of transplantation in 69.8% of cases where time after transplant was reported (**Figure 2**). The most common site was the kidney (N=29), followed by the gastrointestinal tract (N=11), ureter or bladder (N=7), and the perineum (N=7). Mean age (± standard deviation) at time of diagnosis was 49.8 ± 14.8 years; 27 (46%) of patients were male. The most common infectious organism was *E. coli*, present in 42 cases, followed by *Klebsiella* in five cases. Only two cases reported negative bacterial cultures.

**Table 1:** Summary of malakoplakia cases reported in the literature.

N	59
Age [mean (SD)]	49.81 (14.84)
Male Sex (%)	27 (45.8)
Native Disease (%)	
DM/HTN	13 (28.9)
GN	9 (20.0)
Idiopathic	5 (11.1)
Other	7 (15.6)
PKD	3 (6.7)
Recurrent Pyelonephritis	3 (6.7)
Reflux	5 (11.1)
Organ System	
GI (%)	11 (18.6)
Kidney (%)	29 (49.2)
Ureter or Bladder (%)	7 (11.9)
Perineum/Groin (%)	7 (11.9)
Face (%)	4 (6.8)
Abdominal Wall (%)	5 (8.5)
Liver or Spleen (%)	1 (1.7)
Axilla (%)	1 (1.7)
Lung (%)	3 (5.1)
Prostate (%)	1 (1.7)
Organism	
Escherichia coli (%)	42 (85.7)
Klebsiella (%)	5 (10.4)
Enterococcus (%)	3 (6.2)
Proteus (%)	2 (4.2)
Time after Transplant in months [mean (SD)]	35.86 (45.68)
Prior Transplant (%)	3 (5.1)
Immunosuppression Change (%)	
Increased	1 (2.8)
None	5 (13.9)
Reduced	30 (83.3)
Prior Rejection (%)	12 (41.4)
Immunosuppression used	
Tacrolimus (%)	33 (66.0)
Mycophenolate (%)	34 (68.0)
Steroids (%)	41 (82.0)
Cyclosporine (%)	5 (10.0)
Azathioprine (%)	13 (26.0)
Surgical Treatment (%)	
Debridement	7 (12.5)
Resection	13 (23.2)
Percutaneous drainage (%)	7 (12.7)
Malakoplakia Outcome (%)	
Resolution	18 (34.6)
Improvement	26 (50.0)
Persistence	8 (15.4)
Graft Failure (%)	9 (16.7)
Deaths (%)	3 (5.6)

Rejection preceded 41% of cases. The most common immunosuppression agents were corticosteroids (82% of cases where reported), mycophenolate (68%), and tacrolimus (66%), with azathioprine used in 26% of cases. Most cases (83%) were treated by decreasing immunosuppression, 23% with surgical resection, 13% with percutaneous drainage, and 13% with superficial debridement. Three patients died, and malakoplakia improved or completely resolved in 85% of cases. Improvement or resolution was reported in 50% (6/12) of cases where surgical resection was performed, 100% (7/7) of cases with skin-level debridement, and 94% (31/33) of cases where no surgical procedure was performed.

## Discussion

This case series and review of malakoplakia in kidney transplant recipients is the largest do date and highlights three important considerations for timely diagnosis and treatment. First, increased immunosuppression due to rejection or other risk factors should raise suspicion for this condition. Second, malakoplakia is frequently preceded by persistent bacterial infections and challenges with source control, which require long-term antibiotics. Third, most cases of malakoplakia can be managed with lowering immunosuppression and long-term antibiotics, while surgical resection is associated with worse outcomes.

Amongst published cases of malakoplakia, prior rejection was present in 41% of reports that contained this information, an additional three cases had previous transplants, and one case was noted to be “high immunologic risk”. This rate of rejection reported in the literature was higher than the expected rate among kidney transplant recipients (10-20%), suggesting that rejection or the intensified immunosuppression used as part of rejection treatment may be important in the pathogenesis of malakoplakia (4–6). One potential explanation is that higher levels of immunosuppression in these patients could increase the risk of macrophage dysfunction and subsequent malakoplakia.

Early reports suggested a possible association of malakoplakia with use of azathioprine (8, 9). In this review, we found that 26% of patients were taking azathioprine. Many reported cases of malakoplakia occurred in patients taking contemporary immunosuppression including tacrolimus, mycophenolate, and corticosteroids. These results indicate that malakoplakia can occur with a variety of different types of immunosuppression.

Immunosuppression was lowered after the diagnosis of malakoplakia in 83% of cases. This highlights a major gap in the current approach to immunosuppression, which is largely reactive: patients are assumed to be under-immunosuppressed if rejection is detected or over-immunosuppressed in cases of malignancy or infection, including malakoplakia. There is an ongoing need for more personalized strategies to optimize post-transplant immunosuppression regimens (7).

Our analysis also confirms previous analyses that found *E. coli* is a very common infectious source in malakoplakia. While *E. coli* is the culprit organism in 10-65% of all infections in kidney transplant patients, amongst case reports of Malakoplakia *E. coli* was detected in 86%. This suggests that there may be features unique to this organism that increase the risk of malakoplakia. It remains unclear whether infection and inadequate source control induces malakoplakia, or whether the inability to clear infections is an early symptom of malakoplakia. Regardless, challenges in source control should prompt consideration of early cross-sectional imaging and potentially more aggressive antibiotic treatment to prevent development of malakoplakia.

Our findings suggest that the majority of cases resolve or improve with long-term antibiotics and lowering immunosuppression. We also found that surgical debridement is associated with worse outcomes. This is an important finding because malakoplakia frequently first presents as a mass, which might appear amenable to resection. However, the available evidence suggests that resection should not be performed unless absolutely necessary, as long-term antibiotics.

We did not identify any demographic factors associated with case reports of malakoplakia. While a previous review that was not limited to transplant patients suggested a female predominance of malakoplakia (10), in our study 46% of case reports were in male patients, suggesting that the effect of sex on the likelihood of malakoplakia is unlikely to be large.

Limitations of this study include the fact that the available literature is exclusively composed of case reports, which are at high risk of bias due to selective reporting, and some publications do not report core datapoints on risk factors and outcomes. Ideally, future studies would be based on prospective collection of a core set of variables in all cases.

## Conclusions

We report on two cases of malakoplakia and review the literature to characterize risk factors for this rare and challenging condition. We find that malakoplakia most commonly occurs early after transplant and may be preceded by a need for increased immunosuppression, and chronic infections (especially those involving *E. coli*). Early cross-sectional imaging, aggressive antibiotic therapy, and lowering of immunosuppression are the cornerstones of treatment, and a protracted course is expected.

## Data Availability

The original contributions presented in the study are included in the article/**Supplementary Material**. Further inquiries can be directed to the corresponding author.
