# Supplementary material for: Malakoplakia among kidney transplant recipients: case series and literature review
Source: Front Immunol. 2025 May 23;16:1605146. doi: 10.3389/fimmu.2025.1605146 (PMC12140987; doi:10.3389/fimmu.2025.1605146)
Supplement: Supplementary file 1 [file DataSheet1.pdf]

Supplementary Materials

Supplemental Table 1. Search terms derived from previous review.[3]

Source	Search Terms
PubMed	(kidney transplantation[MeSH Terms]) AND (malakoplakia[MeSH Terms])
**Source**	**Search Terms**
PubMed	(kidney transplantation[MeSH Terms]) AND (malakoplakia[MeSH Terms])
Google Scholar	allintitle: ("kidney" OR "renal"), ("transplant" OR "transplantation" OR "allograft"), ("malakoplakia" OR "malacoplakia")
Web of Science	((TI=("kidney" OR "renal")) AND TI=(transplantation OR transplant OR allograft)) AND TI=(malakoplakia OR malacoplakia)
Ovid/EMBASE	(renal or kidney).m_title. AND (transplant or transplantation or allograft).m_titl.AND (malakoplakia or malacoplakia).m title.

Supplementary Table 2: Complete list of Malakoplakia cases in the literature

Link to article	Anatomical Location	Age, Sex	Rejection	Prior transplant	Months after txp	Organism	Immunosuppression	IS change	Antibiotic duration	Surgical/Procedural	**Malakoplakia Outcome**	**Graft outcome**
https://doi-org.ucsf.idm.oclc.org/10.1177/2050313X24	colon	15, M	No	no	18	E. Coli	tacrolimus, mycophenolate	none	1.5 yr	No	**Resolution**	Graft Failure
https://doi.org/10.1053/j.ajkd.2024.01.477	kidney	66, M	No	no		E. Coli	Tacrolimus, mycophenolate mofetil, prednisone. (Tac switched to		NR	No	NR	NR
https://journals.lww.com/jasn/fulltext/2023/11001/a_rare_case_of_malakoplakia_in_a_r	kidney	70, M	No	no	12	Bacillus species	tacrolimus, mycophenolate, and prednisone	reduced	1 mo	No	Persistence	Graft Failure
https://onlinelibrary.wiley.com/doi/10.1111/td.14158	kidney	55, M		no	12	E. Coli	NR	reduced	long term	No	Improvement	Functioning
https://ugeskriftet.dk/videnskab/kutan-malakoplaki	skin (axilla)	48, M		no	12	E. Coli	Mycophenolate mofetil, tacrolimus		6 mo	No	Improvement	Functioning
https://doi-org.ucsf.idm.oclc.org/10.1136/bcr-2023-257130	colon	70s, F		no	156	E. Coli	NR		4 wk + 3 wk	Resection	Improvement	Functioning
https://doi.org/10.23876/j.krcp.22.176	kidney	40, F		no	8	E. Coli	Tacrolimus, mycophenolate mofetil, prednisone	reduced	4 mo	No	Resolution	Functioning
https://doi-org.ucsf.idm.oclc.org/10.1093/ajcp/aqad15	colon	44, M		no		NR	NR		NR	No	NR	NR
https://doi.org/10.1053/j.ajkd.2023.01.408	kidney	43, F		no	7	E. Coli	Mycophenolate, tacrolimus, prednisone	reduced	11 wk	No	Improvement	Functioning
https://www.sciencedirect.com/science/article/pii/S0041134522003955?via%3Dihub	kidney	59, F	Yes	no	18	E. Coli	Tacrolimus, mycophenolate mofetil, prednisone	reduced	longterm	Resection	Improvement	Functioning
https://journals.lww.com/sjkd/fulltext/2023/34040/renal_allograft_malakoplakia_presenti	kidney	49, F	Yes	no	144	E. Coli, later Klebsiella	prednisolone, tacrolimus, and mycophenolate mofetil (at d/c post	reduced	NR	No	Improvement	Functioning
https://www.saudijournals.com/media/articles/SJMPS_62_201-205_c.pdf	kidney	74, F	No	no	24	E. coli, E. aerogenes	tacrolimus, mycophenolic sodium, prednisone	reduced	long-term	No	Resolution	Functioning
https://www.saudijournals.com/media/articles/SJMPS_62_201-205_c.pdf	kidney	62, F	No	no	72	culture negative	tacrolimus, mycophenolic sodium, prednisone	reduced	6 mo	No	Improvement	Functioning
https://www.dustri.com/nc/article-response-page.html?artId=10172&doi=	kidney	23, F	No	no	2	E. coli	NR		28 d	No	Persistence	Graft Failure
https://doi.org/10.1093/ndtplus/sfn028	kidney	56, F	No	yes	11	E. coli	tacrolimus, mycophenolate mofetil, prednisone	reduced	10 wk		NR	Graft Failure
https://doi.org/10.1093/ndt/9.8.1188	kidney, bladder, UVJ	55, F	No	no	36	E. coli	prednisolone, cyclosporine --> azathioprine	reduced	long-term	Resection	Improvement	Functioning
https://doi.org/10.1016/s0046-8177(77)80031-2	kidney	46, F	No	no	15	E. coli, P. vulgaris	prednisone, azathioprine	increased	long-term	Resection	Persistence	Graft Failure
https://doi.org/10.1111/tid.12731	bladder	45, F	No	no	24	E. coli	tacrolimus, mycophenolate, prednisone	reduced	longterm	Resection	Resolution	Functioning
https://jamanetwork.com/journals/jamadermatology/articlepdf/542563/archderm_117_1	skin (surgical site)	52, F	No	no	17	NR	prednisolone, azathioprine, prednisone		6 wk	Resection	NR	NR
https://doi.org/10.3109/003655977091797 0	bladder and allograft	37, F	No	no	6	E. coli, Corynebacterium spp.	azathioprine, prednisolone	reduced	NR	Resection	Persistence	Graft Failure
https://doi.org/10.4132/koreanjpathol.2013.47.2.163	gastrointestinal	55, F	No	no	132	NR	prednisone, tacrolimus, and mycophenolate	none	not used	No	Improvement	Functioning
https://onlinelibrary.wiley.com/doi/epdf/10.1111/j.1432-2277.1999.tb01218.x	abdominal wall, small and large bow-	52, M	No	no	108	E. coli	azothioprine, prednisone, cyclosporin	reduced	NR	Resection	Persistence	Functioning
https://doi.org/10.1136/bcr-2018-227460	abdominal wall	48, M	No	no	5	E. coli	mycophenolic acid, tacrolimus	none	6 wk	No	Improvement	Functioning
https://pubmed.ncbi.nlm.nih.gov/33181747/	kidney	41, F		no	12	culture negative	tacrolimus, mycophenolate mofetil, and corticosteroids		NR		NR	NR
https://link.springer.com/article/10.1007/s10096-021-04270-x	kidney	63, F		no	7	E. coli	tacrolimus, mycophenolate mofetil and prednisolone	none	3 mo	No	Improvement	Functioning
https://link.springer.com/article/10.1007/s10096-021-04270-x	kidney	52, F		no	4	E. coli	tacrolimus, mycophenolate mofetil and prednisolone	reduced	6 mo	No	Improvement	Functioning
https://www.researchgate.net/profile/Xin-Yee-	kidney	55, F		no		E. coli	Mycophenolate, tacrolimus, prednisone	reduced	4 mo	No	Resolution	Functioning
https://www.ajkd.org/article/S0272-6386(18)30469-4/abstract	kidney	36, F		no	48	E. coli	Tacrolimus, mycophenolate mofetil,	reduced	14 wk	No	Improvement	Functioning
https://link.springer.com/article/10.1007/s00467-014-2786-7	kidney	14, F		no	12	E. coli	tacrolimus, mycophenolate mofetil and prednisolone	reduced	3mo	No	Improvement	Functioning
https://doi.org/10.1111/j.1442-2042.2007.01804.x	kidney	45, F		no	24	E. coli	NR		None	Resection	Persistence	Graft Failure
https://www.sciencedirect.com/science/article/pii/S0953620505003559?via%3Dihub	kidney	43, F		no	24	E. coli	mycophenolic acid, tacrolimus		2 mo	No	Improvement	Functioning
https://doi.org/10.1046/j.1365-2559.1996.d01-413.x	kidney	29, F		yes	96	NR	azothioprine, prednisone	none	long-term	No	Improvement	Functioning

Supplementary Table 2: Complete list of Malakoplakia cases in the literature

https://journals.lww.com/asaiojournal/abstract/1994/04000/malacoplakia_of_the_prosta	prostate	60, M		no	12	E. coli, Serratia marcescens	prednisone, cyclosporine	reduced	6 wk	No	Resolution	Functioning
https://doi.org/10.1164/rccm.201511-2134im	pulmonary	67, F		no	12	Rhodococcus equi	mycophenolate		NR	No	Improvement	Functioning
https://pmc.ncbi.nlm.nih.gov/articles/PMC1888084/	pulmonary	44, M		no	31	E. coli	prednisone, azathioprine	reduced	NR	No	Resolution	Functioning
https://doi.org/10.1016/s0190-9622(07)80002-8	skin (temple)	67, M		no	12	E. coli, Streptococcus spp.	azothioprine, prednisone, cyclosporine		5 mo	Debridement	Resolution	Functioning
https://doi.org/10.1016/j.revsto.2015.09.002	submandibular gland, oral mucosa	70, M		no		E. coli	NR		long-term	Resection	Resolution	Functioning
https://doi.org/10.1111/cyt.12086	bladder	64, M		no	36	NR	prednisolone, mycophenolate		NR	No	Improvement	Functioning
https://doi.org/10.1016/0090-4295(85)90270-5	bladder	22, F		no	12	E. coli, P. mirabilis, K.pneumoniae	prednisolone, azathiopurine		NR		NR	NR
https://pmc.ncbi.nlm.nih.gov/articles/PMC6416959/	gastrointestinal, genital (vulva, vagina)	72, F		no	10	NR	mycophenolate, tacrolimus, prednisone		12 wk	Resection	Persistence	Functioning
https://doi.org/10.1038/s41395-018-0382-3	gastrointestinal	51, F		no	132	NR	mycophenolate, tacrolimus, prednisone	reduced	notused	No	Improvement	Functioning
https://journals.lww.com/ajg/fulltext/2017/10001/Colonic_Mass_and_Chronic_Diarrhea	gastrointestinal	68, M		no		NR	NR		6 mo	No	Improvement	Functioning
https://pubmed.ncbi.nlm.nih.gov/16967795/	gastrointestinal	40, M		no	15	E. coli	NR	reduced	6 mo	No	Resolution	Functioning
https://doi.org/10.1590/abd1806-4841.20131790	cutaneous (groin)	51, M		no	24	Providentia spp, C. albicans	NR		NR	Debridement	Resolution	Functioning
https://www.sciencedirect.com/science/article/pii/S1769725521005678?via%3Dihub	groin	70, M		no	24	E. coli, P. aeruginosa	tacrolimus, mycophenolate mofetil, and prednisone		7mo	Debridement	**Resolution**	Functioning
https://www.sciencedirect.com/science/article/pii/S0190962207800028?via%3Dihub	perineum	51, M		no	14	E.Coli, Group G Strep, Klebsiella pneumoniae	methylprednisone, azathioprine		7 weeks	Resection	**Resolution**	Functioning
https://www.sciencedirect.com/science/article/pii/S0190962207800028?via%3Dihub	face, perineum	67, M		no	12	E.coli	azathioprine, cyclosporine, prednisone		None	Debridement	**Resolution**	Functioning
https://pmc.ncbi.nlm.nih.gov/articles/PMC8370553/	kidney	45, F	Yes	no	16	E. coli, K. pneumoniae	tacrolimus, mycophenolate and prednisolone	reduced	long-term	No	Improvement	Functioning
https://pmc.ncbi.nlm.nih.gov/articles/PMC6314856/	kidney	58, M	Yes	no	6	E. coli, E. cloacae	mycophenolate, tacrolimus, prednisone	reduced	1 mo	Resection	Persistence	Graft Failure
https://biomed.papers.upol.cz/artkey/bio-201202-	kidney	31, F	Yes	no	144	E. coli, S. aureus	tacrolimus, mycophenolate mofetil	reduced	NR	No	Resolution	Functioning
https://doi.org/10.1111/tid.12012	perineum	37, M	Yes	no	180	Burkholderia cepacia	tacrolimus, prednisone, mycophenolate sodium		6 mo	No	Resolution	Functioning
https://pmc.ncbi.nlm.nih.gov/articles/PMC1888084/	abdominal wall	32, M	Yes	no	16	E. coli	azothioprine, prednisone,	reduced	NR	Debridement	Improvement	Functioning
https://pmc.ncbi.nlm.nih.gov/articles/PMC1888084/	lung, perianal		No	no	31	E.Coli, Staph Aureus	azothioprine, prednisone,	reduced	9 weeks	Debridement	Resolution	Functioning
https://www.sciencedirect.com/science/article/abs/pii/S0161642086336339	skin (eyelid)	35, M	Yes	no	42	E. coli	NR		NR	No	NR	Graft Failure
https://doi.org/10.1111/nep.12194	bladder and allograft	56, F	Yes	no	12	E coli	tacrolimus, mycophenolate mofetil, prednisone	reduced	12 wk	No	Improvement	Functioning
https://ucsf.primo.exlibrisgroup.com/discovery/openurl?institution=01UCS_SAF&vid=01UCS_SAF:UCSF&volume=115&date=2020&aulast=Ghaith&issn=0002-9270&spage=S870&auinit=J&title=The%20American%20journal%20of%20gastroenterology.&atitle=A%20Case%20of%20Rhodococcus%20Related%20Colonic%20Malakoplakia%20in%20a%20Renal%20Transplant%20Patient&sid=google	gastrointestinal	75, M	Yes	no		NR	tacrolimus, prednisone		3 mo	No	Improvement	Functioning
https://doi.org/10.1097/tp.0b013e3181e7a387	gastrointestinal	45, M	Yes	no	36	NR	tacrolimus and MMF	reduced	2 mo	No	Resolution	Functioning

Supplementary Table 2: Complete list of Malakoplakia cases in the literature

Our case 1	perinpehric	49, F	No	yes	2	E.Coli	tacrolimus, mycophenolate mofetil, and prednisone	reduced	2 years (ongoing)	No	Improvement	Functioning
Our case 2	ureter, bladder, abdominal wall	26, M	Yes	no	4	VRE	tacrolimus, mycophenolate mofetil, and prednisone	reduced	4 months (ongoing)	Debridement	Improvement	Functioning
